# Evaluation of the efficacy of signal-averaged electrocardiogram testing in the cardiac assessment of beta-thalassemia major patients

**DOI:** 10.1186/s12872-022-02984-0

**Published:** 2022-12-07

**Authors:** Maryam Bahmani Jahromi, Amir Hossein Hassani, Mohammad Kasaei, Zahra Hooshanginezhad, Amir Aslani, Omidreza Zekavat, Mohammad Mortezaie, Shahdad Khosropanah

**Affiliations:** 1 Department of Cardiology, Shiraz University of Medical Sciences, Shiraz, Iran; 2 School of Medicine, Shiraz University of Medical Sciences, Shiraz, Iran; 3 Hematology Research Center, Shiraz University of Medical Sciences, Nemazee Hospital, Shiraz, Iran

**Keywords:** Thalassemia major, SAECG, T2*MRI

## Abstract

**Background:**

More than 70% of thalassemia’s major mortality is due to the cardiac complications of this syndrome, mostly consequent to myocardial Iron overload; therefore, evaluation of such complications is of utmost importance. T2*MRI is used to assess hepatic and myocardial Iron load in thalassemia patients, which is not always available. Signal-Averaged Electrocardiography is a rather easy method of evaluating major thalassemia patients regarding their risk for sudden cardiac death.

**Methods and materials:**

In this cross-sectional study, 48 patients with thalassemia major underwent evaluation with electrocardiography, signal-averaged electrocardiography, echocardiography, T2*MRI, and ferritin level. The association of the existence of ventricular late potentials in SAECG and other cardiac variables was evaluated. Moreover, the association between myocardial and hepatic Iron load and cardiac characteristics was assessed.

**Results:**

48 patients with a mean age of 30.31 ± 7.22 years old entered the study. 27 (56.3%) of the patients had ventricular late potentials, which were associated with myocardial dry Iron weight (*P* = 0.011). Nonspecific ST-T changes and premature atrial and ventricular contractions were seen more frequently in patients with late potentials (*P* = 0.002, 0.031, and 0.031, respectively). Patients with higher myocardial and hepatic Iron loads had longer QT_c_ in their 12-lead surface electrocardiograms.

**Conclusion:**

Patients with ventricular late potentials assessed by SAECG had a higher myocardial Iron load. Higher myocardial Iron load is associated with higher cardiac complications in patients with beta-thalassemia major; therefore, SAECG can be used as a screening test for cardiac complications in beta-thalassemia major patients.

## Introduction

Major beta-thalassemia is an inherited hypoproliferative anemic syndrome characterized by hypochromia, microcytosis, and a substantial decline in β chain synthesis that acts as a major concern in healthcare systems around the world [1]. Patients with major thalassemia present with jaundice, dysmorphic musculoskeletal structures, and growth retardation, and they need to receive a tremendous amount of blood transfusions during their lifetime [2]. Long-term infusion of blood products puts the patients at higher risk for Iron overload that deposits in various tissues such as the liver and the heart [3].

Excessive Iron overload in cardiac tissues in major beta-thalassemia patients results in various cardiac complications such as dilated cardiomyopathy, arrhythmias, and congestive heart failure [4]. Such cardiac complications of major thalassemia impose significant risks of morbidity and mortality on the patients to a level that more than 70% of major thalassemia patients die due to cardiac complications of this disease [5, 6].

Hepatic and cardiac Iron overload in major thalassemia patients can be assessed using imaging modalities such as T2* magnetic resonance imaging (T2*MRI) [7]. T2*MRI conduction time has been shown to be indicative of cardiac diastolic function and serum ferritin level [8, 9]. Therefore, T2*MRI can be used to detect cardiovascular complications of major thalassemia. However, this imaging modality is not always accessible and is an expensive diagnostic method.

The Signal-Averaged electrocardiogram is a rather easy method of evaluating major thalassemia patients regarding their risk for sudden cardiac death [10]. Electrophysiologic abnormalities in this population prompt appropriate treatment implementation based on novel findings using modalities such as SAECG, which has long been neglected [11, 12]. Though in this article, we assessed the cardiac function in beta-thalassemia patients with echocardiography and twelve-lead surface electrocardiogram, and we aimed to evaluate the association of SAECG findings in these patients with T2*MRI as an indication of Iron overload.

## Methods and materials

### Ethical statement

This study was conducted in accordance with the Declaration of Helsinki and approved by the Committee of ethics in biomedical research of Shiraz University of Medical sciences by the code IR.SUMS.MED.REC.1400.235. A written consent form was obtained from the patients or their legal guardians.

### Study population

Based on a similar study conducted by Aggarwal et al. considering a type I error of 0.05 and a study power of 80%, using MedCalc Software, the minimal sample size for the study to be statistically significant was 46. The samples were chosen among Major Thalassemia patients referred to the Genetic Research Center of Shahid Dastgheib Hospital affiliated with Shiraz University of Medical Sciences with a random convenience method. The recruitment of the participants lasted from August 2020 to March 2022.

### Eligibility criteria

The inclusion criteria of our study were 10–45-year-old Major Beta-Thalassemia patients who required repeated blood transfusions and Iron chelators from their childhood. The exclusion criteria of our study were: being afflicted with hypertension, diabetes mellitus, gout, ischemic heart disease, cardiac or vascular anomalies (congenital heart disease and valvular disease, among others), and history of active cigarette smoking, any present inflammatory symptoms such as rhinorrhea, cough, pharyngitis, fever, and rhinorrhea, any history of inflammatory diseases such as rheumatoid arthritis and inflammatory bowel disease, the unwillingness of the patients or their legal guardians to participate in the study, and being younger than 10 years old or older than 45 years old.

### Study design

This study followed a descriptive cross-sectional protocol involving 48 MT patients referred to the Genetic Research Center of Shahid Dastgheib Hospital, affiliated with Shiraz University of Medical Sciences. 48 patients with major thalassemia requiring repeated blood transfusions aged between 10–45 years old entered the study. The sampling followed a randomized convenience method.

In this study, a checklist was filled out for each patient after obtaining the written consent form from the patients or their legal guardians. The checklist consisted of five sections. First, demographic information such as age, sex, the frequency of packed cell transfusions, the interval between transfusions, the total number of transfused packed cells, and age at the time of first blood transfusion. The second part of the checklist was attributed to the past medical history of the patients, such as the history of diabetes mellitus, hypothyroidism, adrenal insufficiency, and history of splenectomy, as well as drug history (including Deferiprone, Desfonac, Osfera, and Deferoxamine). The third section involved the information obtained from echocardiography and cardiac Magnetic Resonance Imaging of the patients, such as left ventricular systolic and diastolic function, cardiac chamber sizes, and iron loading state in T2* mapping of liver and heart besides the dry weight of Iron in the heart and the liver. The fourth section of the checklist included the biochemical lab data of the patients as serum ferritin level (Enzyme Immunoassay method, WHO Ferritin 80/602 First International Standard, reference range 15–300 ng/dL), Calcium, 25(OH) Vitamin D_3_, and average pre-transfusion hemoglobin level. The fifth part of the checklist was allocated to electrocardiographic findings (P-wave duration, T-wave duration, P-Q interval, and QT_c_) in a six-minute ECG. Signal Averaged Electrocardiographic findings (Filterd QRS duration, LAS-40, RMS-40) were included in this section.

### Variables and data measurement

Signal‐averaged electrocardiography (SAECG) records low‐amplitude electrical activity in the myocardium. It is used to detect the depolarization of ventricular areas with slow consequences to Iron overload manifested as late potentials (LPs). LPs are described by the duration of the terminal portion of the QRS complex that displays slow amplitude signals (less than 40 μV, known as LAS‐40) and indirectly by the total duration of the filtered QRS complex. Their magnitude is measured by the root mean square voltage of the terminal 40 ms of the filtered QRS complex (RMS‐40). The criteria for diagnosis of LPs proposed in 1991 include (i) filtered QRS duration ≥ 114 ms, (ii) RMS‐40 < 20 μV, and (iii) LAS‐40 > 38 ms [25]. Importantly, these measurements were validated under the strict requirement for the presence of narrow QRS complexes in a standard 12‐lead ECG, thereby excluding a large proportion of patients with right or left bundle branch block or with wide QRS complexes and nonspecific intraventricular conduction defects. To overcome this limitation, modified criteria have been suggested in such patients, encompassing longer duration and lower amplitude of LPs; specifically, (i) filtered QRS ≥ 145 ms, (ii) RMS‐40 < 17.5 μV, and (iii) LAS‐40 > 50 ms can be applied in cases of wide QRS complexes [26]. LPs are generally considered to present when at least 2 of 3 conventional or modified criteria are met.

All of the SAECGs were obtained with “late potential ECG” software from Biomedical Systems revision 5. 0. 3 with an HP Filter of 40–250 Hz and an average beat of 250. HF Noise of less than 0.5 microvolts were considered acceptable. Conventional twelve-lead ECG was also obtained from all patients.

Cardiovascular Magnetic Resonance (CMR) imaging is used as a noninvasive method to evaluate the amount of iron in the heart. MRI examinations were performed with a 1.5 Tesla (T) Magnetom Avanto scanner (Siemens, Erlangen, Germany). The scans included measurement of the liver R2 value and the myocardial T2* value. The scan duration was 45 min. The T2* of the heart was assessed by a cardiac gated single breath-hold multi-echo technique. T2* analysis was conducted using Thalassemia Tools via CVI42 software from Circle company, Calgary, Canada. A full-thickness region of interest (ROI) was drawn in the mid-interventricular septum. The signal intensity of this region for each echo time was measured and plotted as an exponential signal decay curve. Post-processing of acquired images was done by plotting signal intensities and using the curve fitting technique by the CMR42 workstation.

The lower limit of normal for T2* in the detection of myocardial iron deposition has been reported as 20 ms, and this value was used as the cut-off in this study [10]. Patients were divided into 3 groups based on their T2*duration; patients with T2* > 20 ms were considered to be free of cardiac Iron overload, where patients with T2* ≤ 20 ms were deemed to have cardiac Iron overload, and patients with T2* between 10–20 ms were considered to have a moderate Iron overload. Liver MRI scans and cardiac MRI examinations were performed on the same day.

### Statistical analyses

The data were entered into IBM SPSS version 22.0 (Chicago, Illinois, USA). The qualitative data were reported as frequency and percentages, and the quantitative variables were reported as Mean ± SD. T2* was shown as a geometric mean (a log transformation was performed to normalize the data). Correlations between the myocardial T2* value, liver T2* value, echocardiographic parameters, and the serum ferritin level were assessed by Spearman’s rank test. The parameters observed at different levels of T2* and late potentials were compared using one-way ANOVA (analysis of variances). For the comparison of categorical variables, chi-square and Fisher’s exact tests were used, while an independent two-sample T-test was applied for continuous variables. P-values less than 0.05 were considered statistically significant.

## Results

A total of 48 patients with major thalassemia entered the study. 64.6% (31) of the patients were male, and 35.4% (17) were female. The mean age of the participants was 30.31 ± 7.22 years old. The mean age at which transfusions were initiated was 14.94 ± 15.90 months old. The interval between transfusions varied between 14 to 30 days. The mean number of total transfused packed cells in patients was 1077.08 ± 360.30. the average serum ferritin level of the patients was 4020.94 ± 4132.31 ng/ml ranging from 155 to 15,387 ng/ml.

### T2* MRI results

Myocardial and hepatic Iron load were assessed using T2* MRI. In this study, the mean myocardial T2* and hepatic T2* conduction times were 20.29 ± 12.44 and 6.45 ± 6.26 ms, respectively. The findings were equivalent to 2.74 ± 2.55 and 13.99 ± 15.49 mg/g dry Iron weight.

In this study, 25 (52.1%) of the patients had a normal myocardial Iron load (T2*MRI > 20 ms), 6 (12.5%) had moderate myocardial Iron overload (T2*MRI 10–20 ms), and 15 (35.4%) had severe myocardial Iron overload (T2*MRI < 10 ms). 10 (20.8%) patients had a normal hepatic Iron load (T2*MRI > 11.4 ms), while 18 (37.5%) had moderate hepatic Iron overload (T2*MRI 3.3–11.3 ms), and 20 (41.7%) had severe hepatic Iron overload (T2*MRI < 3.3 ms).

### SAECG parameters

All of the patients underwent cardiac assessment by SAECG, six minutes12-lead surface ECG, and Echocardiography. 27 (56.3%) of the patients had ventricular late potentials in their SAECG. 18 (37.5%) of the patients had filtered QRS duration > 114 ms, 16 (33.3%) of the patients had terminal QRS root means square (RMS) voltage < 20 µV, and 27 (56.3%)of the patients had last amplitude (< 40 µV) signal duration of longer than 38 ms.

Table 1 summarizes the demographic, echocardiographic, electrocardiographic, and T2* features of the patient with and without late potentials in their SAECG.

**Table 1:** Comparison of patients with and without ventricular late potentials in their SAECG

Variable		Mean ± SD	*P*-value
*Demographic data*			
Age	Without LP	30.56 ± 6.198	0.795
	With LP	30.00 ± 8.515	
Height	Without LP	163.11 ± 8.396	0.145
	With LP	157.52 ± 17.172	
Weight	Without LP	57.46 ± 8.601	0.226
	With LP	53.91 ± 11.506	
Number of transfused packed cells	Without LP	1140.78 ± 378.985	0.167
	With LP	995.19 ± 325.333	
*Echocardiography*			
Lvedv	Without LP	45.86 ± 7.064	0.688
	With LP	46.73 ± 7.904	
Lvesv	Without LP	26.93 ± 5.247	0.985
	With LP	26.90 ± 7.205	
Lvef	Without LP	58.63 ± 3.559	0.230
	With LP	56.19 ± 9.638	
T2* MRI findings			
Myocardial conduction time	Without LP	24.24 ± 12.183	0.011
	With LP	15.22 ± 11.084	
Myocardial Iron dry weight	Without LP	1.83 ± 2.036	0.009
	With LP	3.85 ± 2.832	
Hepatic conduction time	Without LP	6.74 ± 6.898	0.730
	With LP	6.10 ± 5.479	
Hepatic Iron dry weight	Without LP	14.56 ± 14.241	0.778
	With LP	13.27 ± 17.300	
Ferritin	Without LP	3696.03 ± 4238.336	0.543
	With LP	4438.68 ± 4055.917	
*12-lead surface ECG*			
P-wave duration	Without LP	102.96 ± 33.097	0.572
	With LP	108.48 ± 33.442	
QRS duration	Without LP	109.19 ± 57.105	0.900
	With LP	111.10 ± 44.949	
T-wave duration	Without LP	278.85 ± 37.133	0.625
	With LP	273.90 ± 32.380	
P-Q interval	Without LP	160.89 ± 35.257	0.992
	With LP	161.00 ± 41.469	
Qt_c_	Without LP	443.85 ± 48.600	0.161
	With LP	464.38 ± 50.578	
*SAECG*			
Average beat	Without LP	464.74 ± 84.889	0.709
	With LP	472.95 ± 60.287	
Filter QRS duration	Without LP	96.52 ± 30.393	0.014
	With LP	122.71 ± 40.752	
Duration under 40mv	Without LP	28.74 ± 35.270	< 0.001
	With LP	69.52 ± 34.049	
RMS last 40 ms	Without LP	67.63 ± 54.512	< 0.001
	With LP	11.29 ± 12.511	
H.F noise	Without LP	0.71 ± 1.026	0.350
	With LP	1.09 ± 1.764	

Various abnormalities were found in the electrocardiograms and echocardiograms of the patients. The association between abnormalities such as diastolic dysfunction, premature atrial contractions, and early repolarization, among others, with the existence of Late potentials in the SAECG, are summarized in Table 2. Such associations were assessed by Fisher’s exact test.

**Table 2:** The association of ECG and echocardiographic abnormalities with SAECG late potentials

Variables		With LP	Without LP	P-value
*12-lead surface electrocardiogram*				
Nonspecific ST-T changes	No	10	24	0.002
	Yes	11	3	
PAC	No	17	27	0.031
	Yes	4	0	
PVC	No	17	27	0.031
	Yes	4	0	
IVCD	No	19	25	0.594
	Yes	2	2	
Incomplete RBBB	No	20	25	0.595
	Yes	1	2	
Early repolarization	No	20	26	0.689
	Yes	1	1	
Prolonged QT	No	20	26	0.689
	Yes	1	1	
*Echocardiogram*				
Diastolic dysfunction	No	14	22	0.341
	Yes	7	5	
LA size	Normal	18	26	0.215
	Dilated	3	1	
RA size	Normal	20	27	0.438
	Dilated	1	0	

As can be deducted from Tables 1 and 2, major thalassemia patients with late potentials in their SAECG had higher rates of myocardial Iron overload, nonspecific ST-T changes, PACs, and PVCs. Consequently, SAECG late potentials can be used as a prognostic factor for myocardial Iron overload.

### T2*MRI parameters

Cardiac and hepatic Iron overload was investigated using T2*MRI, and the Iron dry weight of both organs was estimated using this method. Patients were divided into three groups regarding their myocardial Iron load and three groups regarding their hepatic Iron load (normal, moderate, overload, and severe overload). Various electrocardiographic, SAECG, and echocardiographic findings were compared in these groups, which are summarized in Table 3.

**Table 3:** Demographic information, electrocardiographic, echocardiographic and SAECG data of both groups

Variable	Myocardial Iron overload			*P*-value	Hepatic iron overload			*P*-value
	Normal	Moderate	Severe		Normal	Moderate	Severe	
*Demographic information*								
Age	29.96 ± 7.237	30.67 ± 7.763	30.71 ± 7.448	0.942	28.40 ± 8.249	30.94 ± 8.321	30.70 ± 5.695	0.648
Height	160.20 ± 13.769	162.83 ± 6.524	160.59 ± 14.366	0.910	158.70 ± 19.431	159.33 ± 10.353	162.85 ± 11.948	0.627
weight	55.08 ± 7.842	53.92 ± 7.579	57.82 ± 13.357	0.608	56.00 ± 8.718	54.36 ± 10.863	57.25 ± 10.135	0.683
Age at time of first transfusion	16.72 ± 18.17	20.50 ± 12.06	13.18 ± 13.85	0.726	9.40 ± 6.240	16.17 ± 13.461	16.60 ± 20.613	0.473
Transfusion interval	16.96 ± 3.23	16.50 ± 2.81	17.76 ± 4.52	0.698	18.80 ± 3.938	16.56 ± 2.684	16.95 ± 4.110	0.276
Total number of transfused packed cells	1065.44 ± 384.08	981.67 ± 342.28	1127.88 ± 342.18	0.685	943.00 ± 431.514	1045.56 ± 323.070	1172.50 ± 345.772	0.236
Ferritin	3319.12 ± 4387.17	2618.33 ± 1820.74	5548.05 ± 4033.46	0.155	817.81 ± 560.19	3315.88 ± 3350.92	6257.05 ± 4572.86	0.001
*TTE*								
LVEDD	45.23 ± 8.25	46.00 ± 5.76	47.81 ± 6.54	0.546	47.55 ± 8.706	44.45 ± 5.130	47.20 ± 8.377	0.433
LVESD	25.87 ± 5.07	26.50 ± 5.28	28.58 ± 7.58	0.371	25.43 ± 2.565	25.09 ± 4.371	29.30 ± 7.861	0.070
LVEF	59.44 ± 3.52	59.33 ± 2.65	54.29 ± 10.07	0.045	60.50 ± 3.873	58.25 ± 3.735	55.48 ± 9.468	0.151
*12-lead surface ECG*								
P-wave duration	97.36 ± 24.544	108.00 ± 32.069	116.24 ± 41.799	0.189	105.90 ± 32.429	104.06 ± 32.039	106.30 ± 35.718	0.978
QRS duration	106.04 ± 55.142	91.00 ± 16.162	122.59 ± 53.446	0.381	107.30 ± 63.034	99.67 ± 21.920	120.70 ± 63.670	0.457
T-wave duration	266.76 ± 33.784	300.50 ± 51.244	282.88 ± 25.085	0.065	271.10 ± 30.853	281.39 ± 34.326	275.25 ± 38.224	0.742
P-Q interval	151.84 ± 28.461	181.50 ± 37.612	167.06 ± 46.741	0.159	161.60 ± 38.679	153.94 ± 28.532	166.90 ± 44.612	0.580
QTc	428.44 ± 24.947	443.67 ± 27.790	491.94 ± 59.883	< 0.001	424.70 ± 20.602	447.39 ± 44.423	471.80 ± 58.283	0.040
*SAECG*								
Average beat	470.80 ± 65.267	420.00 ± 44.077	481.76 ± 90.714	0.215	433.00 ± 60.606	475.67 ± 55.225	479.40 ± 91.779	0.243
Filter QRS duration	103.48 ± 30.834	100.00 ± 18.783	117.41 ± 48.961	0.431	109.90 ± 25.243	115.11 ± 41.730	100.60 ± 38.359	0.490
LAS-40	38.28 ± 29.724	53.00 ± 66.630	56.53 ± 41.924	0.326	47.00 ± 26.285	56.39 ± 52.381	37.55 ± 31.582	0.357
RMS-40	49.96 ± 44.588	42.33 ± 24.809	32.94 ± 63.327	0.567	47.50 ± 61.031	32.94 ± 29.769	49.75 ± 59.103	0.567
HF noise	0.706 ± 1.0692	0.414 ± 0.1475	1.289 ± 0.8760	0.289	0.491 ± 0.4596	0.938 ± 1.3227	1.0131 ± 1.7437	0.619

Pearson’s correlation test was used to assess the relationship between echocardiographic, electrocardiographic, T2*MRI, and SAECG findings. Myocardial and hepatic dry Iron weight had significantly positive correlations with QT_c_ (r = 0.368, *P* = 0.010, and r = 0.317, P0.028, respect. Therefore, myocardial and hepatic T2*MRI conduction times had significant inverse relationships with QT_c_ (r = −0.574, *P* < 0.001, and r = −0.358, *P* = 0.012, respectively). Ferritin level had a significant correlation with hepatic dry Iron weight (r = 0.421, *P* = 0.003) but not myocardial dry Iron weight (r = 0.257, *P* = 0.078). among SAECG findings, myocardial dry Iron weight had a significant direct correlation with LAS-40 (r = 0.304, *P* = 0.036). moreover, the duration of the filtered QRS wave and P–R interval showed a significant and direct correlation (r = 0.297, *P* = 0.040). the results of Pearson’s correlation tests are summarized in Table 4.

**Table 4:** Assessment of the correlations between SAECG, T2*MRI, electrocardiographic, and echocardiographic findings

	LVEDV	LVESV	Myocardial T2*MRI	Myocardial dry Iron weight	Hepatic T2*MRI	Hepatic dry Iron weight	Ferritin	P duration	QRS duration	T duration	PR interval	QT_c_	Average beat	Filtered QRS	LAS-40	RMS-40
LVEDV	1															
LVESV	R = 0.380 *P* = 0.008	1														
Myocardial T2*MRI	R = −0.124 *P* = 0.402	R = −0.289 *P* = 0.046	1													
Myocardial dry Iron weight	R = 0.087 *P* = 0.558	R = 0.112 *P* = 0.449	R = −0.799 *P* < 0.001	1												
Hepatic T2*MRI	R = 0.078 *P* = 0.597	R = −0.255 *P* = 0.080	R = 0.414 *P* = 0.003	R = −0.372 *P* = 0.009	1											
Hepatic dry Iron weight	R = 0.052 *P* = 0.726	R = 0.272 *P* = 0.061	R = −0.248 *P* = 0.089	R = 0.245 *P* = 0.093	R = −0.614 *P* < 0.001	1										
Ferritin	R = 0.050 *P* = 0.735	R = 0.011 *P* = 0.939	R = −0.225 *P* = 0.125	R = 0.257 *P* = 0.078	R = −0.492 *P* < 0.001	R = 0.421 *P* = 0.003	1									
P duration	R = −0.152 *P* = 0.303	R = = −0.121 *P* = 0.412	R = −0.280 *P* = 0.054	R = 0.264 *P* = 0.070	R = −0.031 *P* = 0.834	R = 0.159 *P* = 0.279	R = −0.087 *P* = 0.554	1								
QRS duration	R = −0.040 *P* = 0.786	R = −0.043 *P* = 0.770	R = −0.077 *P* = 0.604	R = 0.100 *P* = 0.498	R = −0.044 *P* = 0.768	R = 0.259 *P* = 0.075	R = 0.003 *P* = 0.986	R = 0.538 *P* < 0.001	1							
T duration	R = 0.006 *P* = 0.966	R = 0.163 *P* = 0.267	R = −0.372 *P* = 0.009	R = 0.296 *P* = 0.041	R = −0.137 *P* = 0.352	R = −0.021 *P* = 0.889	R = −0.131 *P* = 0.374	R = 0.333 *P* = 0.021	R = −0.094 *P* = 0.527	1						
PR interval	R = 0.082 *P* = 0.582	R = −0.044 *P* = 0.767	R = −0.247 *P* = 0.090	R = 0.165 *P* = 0.262	R = −0.074 P = 0.617	R = 0.176 P = 0.231	R = −0.102 P = 0.492	R = 0.742 *P* < 0.001	R = 0.158 *P* = 0.284	R = 0.413 *P* = 0.004	1					
QT_c_	R = 0.104 *P* = 0.484	R = 0.267 *P* = 0.066	R = −0.574 *P* < 0.001	R = 0.368 *P* = 0.010	R = −0.358 *P* = 0.012	R = 0.317 *P* = 0.028	R = 0.195 *P* = 0.184	R = 0.506 *P* < 0.001	R = 0.297 *P* = 0.040	R = 0.340 *P* = 0.018	R = 0.389 *P* = 0.006	1				
Average beat	R = 0.034 *P* = 0.819	R = 0.135 *P* = 0.362	R = −0.016 *P* = 0.914	R = −0.037 *P* = 0.803	R = −0.157 P = 0.285	R = 0.231 *P* = 0.114	R = 0.458 *P* = 0.001	R = −0.023 *P* = 0.875	R = 0.116 *P* = 0.434	R = −0.375 *P* = 0.009	R = −0.156 *P* = 0.291	R = 0.187 *P* = 0.204	1			
Filtered QRS	R = 0.021 *P* = 0.887	R = −0.181 *P* = 0.218	R = −0.151 *P* = 0.305	R = 0.206 *P* = 0.160	R = 0.042 *P* = 0.779	R = −0.246 *P* = 0.092	R = 0.075 *P* = 0.611	R = 0.330 *P* = 0.022	R = 0.152 *P* = 0.302	R = −0.133 *P* = 0.367	R = 0.297 *P* = 0.040	R = 0.279 *P* = 0.054	R = 0.030 *P* = 0.838	1		
LAS-40	R = 0.213 *P* = 0.146	R = 0.041 *P* = 0.783	R = −0.253 *P* = 0.083	R = 0.304 *P* = 0.036	R = 0.020 *P* = 0.895	R = −0.127 *P* = 0.388	R = 0.001 *P* = 0.955	R = −0.121 *P* = 0.414	R = −0.166 *P* = 0.260	R = −0.047 *P* = 0.749	R = −0.035 *P* = 0.815	R = 0.019 *P* = 0.897	R = −0.011 *P* = 0.939	R = 0.373 *P* = 0.009	1	
RMS-40	R = −0.085 *P* = 0.564	R = −0.056 *P* = 0.704	R = 0.177 *P* = 0.228	R = −0.207 *P* = 0.158	R = 0.008 *P* = 0.957	R = 0.084 *P* = 0.570	R = −0.062 *P* = 0.674	R = 0.022 *P* = 0.879	R = 0.019 *P* = 0.897	R = −0.010 *P* = 0.949	R = 0.015 *P* = 0.919	R = -0.191 *P* = 0.020	R = −0.336 *P* = 0.020	R = −0.220 *P* = 0.133	R = −0.427 *P* = 0.003	1

## Discussion

In this study performed on 48 major thalassemia patients, we found that the mean hepatic and myocardial T2*MRI conduction times were 20.29 ± 12.44 and 6.45 ± 6.26 ms, respectively. Patients with ventricular late potentials in their SAECG tended to have lower myocardial T2*MRI conduction times and, thus, have higher myocardial Iron loads. Patients with ventricular late potentials in their SAECG also had higher rates of non-specific ST-T changes, Premature atrial contraction, and Premature ventricular contractions. Moreover, patients with higher myocardial and hepatic Iron load had longer QT_c_. The application of T2* MRI in defining tissue Iron load in thalassemia patients has long been proven [13, 14]. Therefore, in this study, we used this relatively novel method in defining the Iron load of the cardiac and hepatic tissues in beta-thalassemia major patients to see if iron overload is related to changes in electrocardiography, SAECG, and echocardiography of the patients.

Daar et al. have found that lower cardiac T2* in thalassemia major patients is associated with higher mortality; consequently, further evaluation of patients with lower cardiac T2* is of great importance [15]. Moreover, since T2* MRI is not readily available in most institutes, finding proper substitutes for this modality is also essential.

In our study, although ferritin level was associated with hepatic Iron load, it did not have any association with the cardiac Iron load. This finding was in line with the findings of a study performed by Khadivi Heris et al. on 58 thalassemia major patients [16]. They also found that cardiac Iron load was associated with higher Alanine Aminotransferase levels, which was not evaluated in our study. On the other hand, in a study by Wahidiyat et al., they found that ferritin level was associated with cardiac and hepatic T2*MRI conduction times [17].

In a study by Dursun et al., the authors found that lower cardiac T2* MRI conduction time was associated with a larger interventricular septum and higher left ventricular mass, which can be due to iron deposition in the cardiac tissue [18]. However, no association was found between T2* MRI conduction time and left ventricular ejection fraction. However, in our study, patients with higher myocardial iron load had lower left ventricular ejection fractions and were more in danger of congestive heart failure, which shows that iron overload in cardiac tissues induces systolic and diastolic dysfunction.

In a study by Aggarwal and colleagues performed on 48 thalassemia major patients, higher cardiac Iron overload was associated with longer QT_c_ in the patients [19]. This finding was in line with the results of our study, indicating that a higher Iron load in beta-thalassemia major patients is accompanied by higher cardiovascular mortality.

In a study by Hayıroğlu et al., they used a formula devised from 12-lead ECG findings to predict the existence of diastolic dysfunction in patients. Such findings can also be extremely useful in the population of patients with major thalassemia since most of the patients with cardiac Iron overload have diastolic dysfunction, which might end in further complications such as heart failure with preserved ejection fraction and ultimately sudden cardiac death [20].

In this study, we also assessed the patients using signal-averaged electrocardiography since T2* MRI is not always available, but recording SAECG is a rather easy way of ventricular function assessment. In our study, patients with ventricular late potentials assessed by SAECG were found to have higher cardiac Iron load and, thus, lower cardiac T2* MRI conduction time. However, the parameters that were significantly different among patients with variable degrees of cardiac Iron load, such as, QT_c,_ did not differ in patients with and without ventricular late potentials. This might indicate that ventricular late potentials have higher sensitivity and lower specificity for cardiac abnormalities such as QT prolongation; therefore, this method can be used as a screening tool for cardiac complications of beta thalassemia major.

In our study, myocardial and hepatic dry Iron weight had direct and significant correlations with QT_c_ and T duration, which indicates the prolongation of the ventricular repolarization phase. Since QT_c_ prolongation is associated with higher mortality, further studies to understand the underlying mechanism of this finding and its importance seem essential [21].

In a study conducted by Patsourakos et al., the existence of ventricular late potentials in SAECG was associated with longer QRS duration, longer QT interval, larger left ventricular end-diastolic diameter, larger left atrial volume index, and higher pulmonary artery systolic pressure, all indicating more severe cardiac complications of beta thalassemia major [11]. In our study, non-specific ST-T changes, premature atrial contractions, and premature ventricular contractions were seen more often in patients with ventricular late potentials, however, left atrial size and left ventricular end-diastolic volume did not have a significant association with ventricular late potentials. Since the patients in Patsourakos’ study were older than our subjects, these findings might indicate that patients with ventricular late potentials are at higher risk for mentioned complications, such as left atrial enlargement, ventricular hypertrophy, and diastolic dysfunction in the long term.

In a study by Franzoni et al., thalassemia major patients underwent Holter ECG monitoring, and patients with ventricular late potentials showed higher rates of ventricular tachycardia, which shows that this method can be used as a screening method for cardiac complications of beta thalassemia major. This is in line with the results of our study that patients with ventricular late potentials in their SAECG need further cardiac evaluations [22].

Isma’eel et al. also evaluated 26 patients with beta-thalassemia major for seven years and assessed them with SAECG. In this period, ferritin levels were correlated with QRS duration and RMS voltage. Therefore, they concluded that these two SAECG parameters could be used as an indicator of Iron overload and cardiac complications [12].

In our study, filtered QRS duration was significantly correlated with the P–R interval. Although atrioventricular blocks are rare in thalassemia major patients and only a few case reports exist in this matter [23], prolonged filtered QRS in the patients might act as a prognostic factor for the development of this complication. Animal studies have revealed the effect of Iron overload in slowing the conduction of impulses in cardiomyocytes which prompts further research into this matter [24].

## Conclusion

In our study, we found that patients with ventricular late potentials assessed by SAECG had a higher myocardial Iron load, and a higher myocardial Iron load is associated with higher cardiac complications in patients with beta-thalassemia major. However, the changes in cardiac function occur in a long term. Consequently, SAECG can be used as a screening test for cardiac complications in beta-thalassemia major patients.

## Data Availability

All the data analysed during the current study are included in the published article. The dataset generated and analyzed during the current study are not publicly available due to the patient’s privacy, but are available upon reasonable request from the corresponding author.

## Abbreviations

T2*MRI: T2* magnetic resonance imaging
CMR: Cardiovascular magnetic resonance
ECG: Electrocardiography
SAECG: Signal‐averaged electrocardiography
LPS: Late potentials
ms: Milliseconds
